# Un cas de spondylodiscite d’Anderson

**DOI:** 10.11604/pamj.2020.36.332.19979

**Published:** 2020-08-24

**Authors:** Kaba Condé, Garba Mahaman Salissou

**Affiliations:** 1 Service de Rhumatologie, Centre Hospitalier Universitaire Ignace Deen, Conakry, Guinée,; 2 Service de Rhumatologie, Centre Hospitalier Régional de Maradi, Maradi, Niger

**Keywords:** Spondylodiscite d’Anderson, spondylarthrite ankylosante, Anderson's spondylodiscitis, ankylosing spondylitis

## Abstract

We report the case of a 48-year old patient, with no particular previous history, on follow up for ankylosing spondylitis according to ASAS (Assessment of SpondyloArthritis international Society) criteria since 2012. The patient had inflammatory lower back pain without triggers fever, cough or weight loss. Physical examination showed lumbar stiffness with Schöber index 10+1, fingertip-to-floor distance = 30 cm. X-ray was not contributory. Lumbar MRI objectified Anderson spondylitis at L4-L5. These data were confirmed by lumbar CT scan, which showed spondylolysis at L4-L5 with erosions. Infection or neoplasm were excluded causes of Anderson’s spondylodiscitis. Patient’s outcome was favorable under analgesic, anti-inflammatory treatments and lumbar belt.

## Image en médecine

Nous rapportons le cas d’un patient de 48 ans sans antécédent particulier suivi pour une spondylarthrite ankylosante depuis 2012 en accord avec les critères d’ASAS (Assessment of SpondyloArthritis International Society). Il présente une lombalgie d’horaire inflammatoire sans facteurs déclenchants. Il n´était retrouvé ni notion de fièvre, ni de toux, ni d’amaigrissement. L’examen physique montrait une raideur lombaire avec l’indice de Schöber 10+1, la distance doigts-sol = 30 cm. La radiographie n´était pas contributive. L’imagerie par résonance magnétique (IRM) lombaire révèle une spondylite d’Anderson au niveau L4-L5, ces données sont confirmées par le scanner lombaire qui met en évidence une spondylolyse avec érosions L4-L5. Une cause infectieuse et néoplasique a été éliminée. L´évolution était favorable sous traitement antalgique, anti-inflammatoires et port de ceinture lombaire.

**Figure 1:**
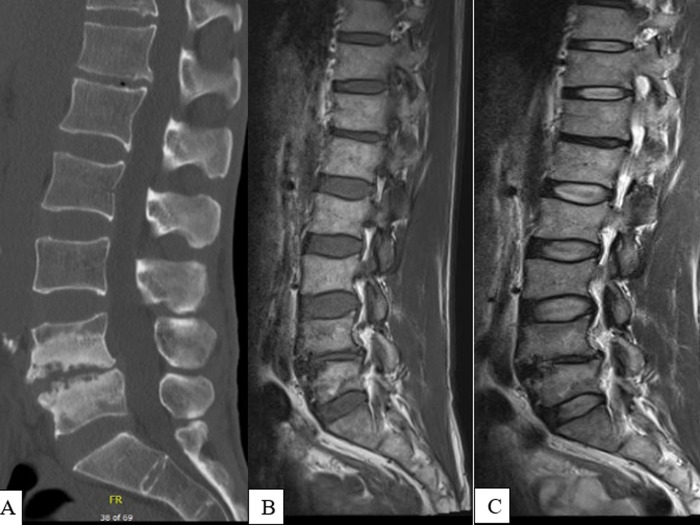
cas de spondylodiscite d’Anderson

